# Young Children's Understanding of Restorative Justice

**DOI:** 10.3389/fpsyg.2021.715279

**Published:** 2021-09-28

**Authors:** Zheng Zhou, Wan-chi Wong

**Affiliations:** ^1^Research Institute of Social Development, Southwestern University of Finance and Economics, Chengdu, China; ^2^Department of Educational Psychology, The Chinese University of Hong Kong, Shatin, Hong Kong, SAR China

**Keywords:** restorative justice, retributive justice, moral transgression, early moral development, Representational Redescription model, choice-based paradigm

## Abstract

The present study investigated how young children understand the sophisticated concept of restorative justice in unintentional moral transgressions. A sex-balanced sample of 5-year-old (*M* = 5.67, *SD* = 0.34, 49.3% girls) and 8-year-old (*M* = 7.86, *SD* = 0.29, 46.0% girls) Chinese children (*N* = 193) participated in the study. In designing the materials, we distilled the multidimensional meanings of restorative justice into two stories, one addressing the theme of property violation and the other physical harm; both stories were set in an animal community. We then engaged the children in joint reading and an interview, during which they showed preference for the given treatments for the transgressor (two restorative treatments vs. two retributive treatments) and ranked two further sets of restorative vs. retributive treatments at the community level. The results indicated that most children favored restorative treatments over retributive treatments for a transgressor, and the 8-year-olds viewed psychological restoration more favorably and behavioral punishment less favorably than the 5-year-olds. The children also tended to endorse restorative treatments at the community level, revealing an understanding of the needs, and obligations of all parties concerned. Notably, more 8- than 5-year-olds showed a consistency in restorative orientation at this level. Interpreting our data through the lens of the Representational Redescription model, we attained a more refined account of young children's levels of understanding regarding restorative justice. These results provide insights for the early cultivation of restorative justice among young children, which is a cornerstone for its successful practice in any society.

## Introduction

The means to achieve justice in responding to moral transgressions has been a matter of debate for thousands of years. Generally, there are two distinct paradigms of response to wrongdoing: retributive/punitive justice and restorative justice. Retributive justice emphasizes the punishment of wrongdoers and has long been the primary practice in the legal system. However, retributive justice is now criticized for destroying people's social personality (Consedine, 1995), fueling conflict, and deepening harm, especially for relatively minor offenses (Daly and Immarigeon, 1998; Zehr, 2002/2015). In recent decades, restorative justice is regarded as a meaningful solution to the excessive reliance on punishment (Barnett, 1977), and its value has been increasingly recognized globally (see Sullivan and Tifft, 2006; van Wormer and Walker, 2013).

Restorative justice concerns healing the harm caused by wrongdoing and meeting the needs of the involved parties, including the victims, offenders, and communities (Van Ness and Strong, 1997/2015; Daly, 2000; Zehr, 2002/2015). Despite the increasing importance of restorative justice in jurisprudence (see Braithwaite, 2002a,b) and its application extending from the legal system to peacemaking circles, school systems, and family group conferencing (see Strang and Braithwaite, 2001; Sullivan and Tifft, 2006; van Wormer and Walker, 2013), surprisingly little research has focused on how children understand the concept of restorative justice compared to the vibrancy of research on children's understanding of moral concepts such as distributive justice (e.g., Fehr et al., 2008; LoBue et al., 2011; Smith and Warneken, 2016) and procedural justice (e.g., Gold et al., 1984; Shaw and Olson, 2014). The current study addressed the gap in the literature by investigating young children's preference for restorative treatment or punitive treatment in response to varied moral transgressions.

Research on early moral development has flourished in recent decades. A special issue in Human Development featured the state of the art of this research field (Smetana, 2018a). In the introductory essay to this special issue, Smetana (2018b) focused the discussion on the advanced moral capacity of infants and young children shown by various research programs. In the commentary that served as the final paper, Turiel (2018) insightfully remarked that the new findings on moral capacity in the early years are not in line with the influential moral formulation in the field, in which developmental sequences culminate in autonomous morality (postulated by Piaget) and stages of principled morality (postulated by Kohlberg) in a much later period. He further raised the issue of universality vs. cultural specificity in this thriving field of study (Turiel, 2018). This special issue presents readers with diverse theoretical propositions and methods used in the research endeavors of early moral development.

Within the field of early moral development, there is a research focus on the emergence of the sense of justice, particularly young children's understanding of distributive justice. In an experimental study on egalitarianism in young children, Fehr et al. (2008) found that most children aged 7–8 preferred resource allocation that removed advantageous or disadvantageous inequality, while the behaviors of those aged 3–4 were characterized by self-interest. Notably, a research team at the Max-Planck Institute for Evolutionary Anthropology found that children as young as 3.5 and 4.5 years of age were capable of showing an aversion to disadvantageous and advantageous inequities (Ulber et al., 2017). A cross-sectional study of resource allocation conducted among 3-, 5-, and 8-year-olds further revealed that young children's understanding of distributive justice developed from an equality preference to an acceptance of legitimate reasons for unequal allocation of resources, which included the consideration of merits, needs, and agreed-upon rules (Schmidt et al., 2016).

An earlier study on children's understanding of procedural justice was conducted in the context of their reaction to authorities' decisions regarding punishment (Gold et al., 1984). The results indicated that both first and fifth graders were sensitive to the manipulations of procedural justice. Shaw and Olson (2014) conducted a series of experiments on young children's preference for partial vs. impartial procedures in the context of resource distribution. In a sample of 5- to 8-year-olds, the older children demonstrated a stronger aversion to the use of partial procedures, suggesting an increasingly positive valuation of procedural justice in middle childhood. Through the research design of a real-life allocation activity in small groups, Xu and Wong (2014) investigated Chinese children's understanding of procedural justice along the implicit-explicit spectrum. The mastery of procedural justice among 5- to 9-year-old children was found to be relatively low, as reflected by both behavioral performance and verbal explanation at the individual level. Based on the assessment indices for group performance developed from the procedural perspective of Habermas (1983/1990), the 7-year-old group showed a significantly enhanced implicit understanding of procedural justice compared with the 5-year-old group.

Young children's reactions to norm violation and their understanding of punitive justice constitute a further research interest in the field. It was found that children as young as 3 years of age exhibited normative responses such as protest, critique, and teaching when encountering a mistake made by a puppet in conventional games (Rakoczy et al., 2008). Three-year-olds also tended to protest when a third party's property rights were violated (Rossano et al., 2011). Past research has also suggested that young children have a sense of what and why proper treatments should be adopted in responding to wrongdoing. Piaget (1932/1997) found that younger children in the egocentric stage selected more severe punishments than older children. Researchers in developmental and evolutionary psychology have further investigated children's use of punishment in different contexts and its rationale (e.g., Helwig et al., 2001; Salali et al., 2015; Smetana and Ball, 2018). A study conducted by Marshall et al. (2021) further investigated the retributive and consequentialist motives of children in using punishment. However, children's endorsement of restorative treatment, which serves to heal the harm done to the victim and the community, has largely been a neglected research area.

A pioneering study on restorative justice in young children was conducted by Riedl et al. (2015). Through the special design of a turnable table, the researchers applied an innovative action-based paradigm to examine the respective punitive and restorative responses of young German children in an experimental setting. The results of the first experiment indicated that both the 3- and 5-year-old children tended to remove the toy or food away from a puppet, who had grabbed the item away from its owner. A further experiment found that children as young as 3 years of age tended to return the toy or food to the original owner, among other options, when the item was grabbed away by a puppet. In both experiments, children showed the tendency to intervene in a violation where they were a third-party witness just as they did in the case where they were personally affected in a second-party condition. Riedl et al. (2015) interpreted such behavioral responses as reflecting a sense of justice, which might be attributable to an understanding of the harm caused to the victim. There have been emerging interests in children's punishment and restoration among researchers most recently. Notably, McAuliffe and Dunham (2021) found that 6- to 9-year-olds in an American sample favored punishment over restoration. However, Yang et al. (2021) found that 3- to 6-year-old Chinese children preferred restoration to punishment in both the roles of second-party victims and third-party bystanders, with older children showing a stronger preference for restoration than the younger ones while they were victims of transgressions.

It is worthy to note that the above-mentioned pioneering studies on restorative justice have not yet taken the multidimensional meanings of restorative justice into account. Recognizing that the restorative conception of justice has its roots in both Western and non-Western traditions, some of its proponents have regarded the contemporary discourse and practice as a revival of old traditions (Llewellyn and Howse, 1998; Johnstone, 2001/2011). Eglash is credited with coining the term “restorative justice” in his article entitled “Beyond restitution: Creative restitution” (Eglash, 1977). In Eglash's conceptualization, the concern of creative restitution or restorative justice primarily lies in recognizing the harm caused by the offense and considering the victim's needs (Eglash, 1958, 1977). Zehr, generally regarded as the grandfather of the contemporary restorative justice movement, provided important clarification of the multidimensional meanings of restorative justice. With respect to the restorative process, Zehr (2002/2015) highlights the identification of three major stakeholders, namely, the victim, the offender, and the community. With its aim of righting wrongs and harms, Zehr (2002/2015) proposes three central concepts or pillars of restorative justice. The first pillar constitutes the harms and related needs that involve the victim, the offender, and the community; the second pillar concerns itself with the obligations caused by the harms; the third pillar involves the engagement of all concerned parties in the justice-seeking process. The multidimensional meanings of restorative justice clarified by Zehr have been embraced by subsequent discourses in the field (see Van Ness and Strong, 1997/2015; van Wormer and Walker, 2013).

Although research on restorative justice in developmental psychology is only a recent endeavor, studies related to restorative and retributive treatment have been conducted. A typical psychological restoration to alleviate the harms caused by wrongdoing is an apology. There is evidence that children aged 4–9 years could have a basic understanding of the emotional effects of apology on a transgressor and a victim (Smith et al., 2010). A recent study found that children as young as 4 years of age were more forgiving of a transgressor who had apologized than one who had not, and 5-year-olds were more forgiving of a remorseful wrongdoer than an unremorseful wrongdoer even when the wrongdoer did not explicitly apologize to the victim (Oostenbroek and Vaish, 2019).

In contrast to a verbal apology, actual or behavioral restoration provides the victim with actual compensation for the harm or loss. Transgressors performing actual restitution are believed to express a greater commitment to rectifying their wrongdoings than those offering a mere apology (Carlisle et al., 2012). Drell and Jaswal (2016) found that 6- to 7-year-olds' negative feelings decreased when an offender offered behavioral restitution. A study focusing on college students also suggested that restitution enhances forgiveness (Carlisle et al., 2012).

Unlike restorative justice, retributive justice focuses on punishing an offender (Daly, 2000). It is worth noting that previous research mostly asked a general and abstract question about how much punishment the offender deserved (see Cushman, 2008; Jambon and Smetana, 2014; Smetana and Ball, 2018). Research that differentiates and compares children's judgment on psychological and actual punishment is lacking.

When the community's role is taken into consideration, treatments for a wrongdoer can take additional forms, such as exclusion and education. Although children in one study considered it generally morally wrong to exclude others from the group, exclusion was regarded as more acceptable if it was done for the sake of group norms and group functioning (Killen and de Waal, 2000). In another study, exclusion was also endorsed by children in the context of a member's unequal distribution of resources in a group (Hitti et al., 2014). A line of multidisciplinary research has endeavored to examine, in contrast with exclusion as a punitive response to transgression, the creation of inner and outer spaces for making changes to attain restorative justice (see Gavrielides, 2015).

Given the limited ability of young children to express themselves through language, investigating their understanding of a sophisticated justice concept, such as restorative justice, is a challenging task. However, such analyses are possible by interpreting young children's preferences for restorative treatments vs. retributive treatments through the lens of the Representational Redescription model (RR model). The RR model postulates that the acquisition of concepts and knowledge is achieved at different levels along a spectrum of the implicit-explicit dimension (Karmiloff-Smith, 1992/1999). Specifically, four levels of representation are postulated along the spectrum, namely, Implicit (I), Explicit-1 (E1), Explicit-2 (E2), and Explicit-3 (E3). At the implicit level (Level I), children represent knowledge in procedural form, and interdomain representational links are not yet developed. Thus, the behaviors generated by implicit understanding appear inflexible. At Level E1, representation is more cognitively flexible, and such flexibility could be reflected in children's consistency in performance across domains based on their understanding of a certain concept. However, the representations at Level E1 are not yet consciously accessed until Level E2 is reached. Level E3, which is considered the most explicit level of understanding, is characterized by the ability to verbally articulate representations. This RR model has been applied to explain concept and knowledge acquisition in the domains of linguistics, physics, mathematics, notation, and theory of mind (see Karmiloff-Smith, 1992/1999). In recent years, young children's understanding of distributive justice and procedural justice was further examined through the lens of the RR model (Xu and Wong, 2014, 2016).

The RR model could also serve as a valuable lens in examining young children's understanding of restorative justice. In the case that a child can indicate a preference for a restorative option in a moral transgression scenario, he or she might have understood the concept at an implicit level. If he or she consistently prefers restorative choices across different situations in response to a moral transgression, this behavior could be interpreted as demonstrating an understanding of restorative justice at least at Level E1, which according to the RR model, is the initial stage of explicit understanding. When a clear explanation of the meaning of restorative justice is given by a child as a justification for his or her choices, this indicates that the child has reached Level E3, a high level of explicit understanding at which representations are conscious and can be verbally articulated. Level E2, which is characterized by representations that are consciously accessible but not yet verbally articulable, is difficult to detect in empirical studies. Hence, Karmiloff-Smith (1992/1999) names E2 and E3 collectively E2/3 in the research context.

In adherence to the RR model, the current study applied a choice-based paradigm and integrated it with consistent analysis of restorative orientation. This is a unique characteristic of this study, and the specific details are subsequently discussed.

While the practice of restorative justice has been gaining momentum in the legal system and social institutions of occidental countries in recent decades (Johnstone, 2001/2011; Sullivan and Tifft, 2006; van Wormer and Walker, 2013), how children understand restorative justice is still unclear. Because the successful practice of restorative justice relies much on the endorsement and engagement of the community as a whole, the cultivation of the values of restorative justice becomes important. In this light, an investigation into how young children make sense of restorative justice serves as a fundamental step by which the preconditions for cultivating the restorative orientation could be uncovered. Though some recent studies have been interested in children's restorative behavior, no research has investigated children's preference for restorative justice at both individual level and community level. The current study originated from a desire to capture young children's understanding of the multidimensional meanings of restorative justice in a possibly comprehensive way, which the abovementioned experimental studies might not achieve. Its uniqueness lies in the design of an interactive story-reading activity, through which the researchers could distill the key components of restorative justice in the created scenarios of moral transgressions and make them comprehensible to young children.

Applying a choice-based paradigm embedded in interactive story reading, the overarching goal of the current study is to investigate young children's understanding of restorative justice in unintentional moral transgression scenarios. The intricate roles of intentionality and harmful consequences in the moral judgment exercised by different age groups have been well-documented in the literature (Piaget, 1932/1997; Yuill and Perner, 1988; Zelazo et al., 1996). In full recognition of the complexity of restorative justice and the different possibilities of studying it from the perspective of developmental psychology, in the current study, we first chose to focus on examining young children's understanding of this multidimensional concept in the context of unintentional actions with harmful consequences.

The first research aim of the study lies in examining young children's preference for restorative treatment vs. retributive treatment with regard to two unintentional moral transgressions, one involving a property violation and the other involving physical harm. Children randomly assigned to read one of the stories were asked to rank their preference regarding the four treatments of the transgressor, two of which were restorative, and two of which were retributive. For each treatment type, one psychological treatment and one actual or behavioral treatment were designed. Based on the results of the pioneering study on children's sense of restorative justice (Riedl et al., 2015), we expected that young children would be in general capable of showing a preference for restorative treatments in unintentional moral transgressions.

The second research aim of the current study is to investigate whether the respective hardship backgrounds of the victim and the transgressor affect young children's treatment preference ranking. To investigate this issue, two rounds of treatment ranking were built into the design of the interactive story reading, one before and one after the introduction of the hardship background of either the victim or the transgressor. The consistency in children's restorative orientation despite the manipulation of the hardship background of the involved parties will be assessed in light of the RR model. Considering Hoffman's (1990) thesis that empathic bias could have an impact on the justice-seeking process, we expected that young children's treatment rankings regarding the transgressor to be affected due to the empathy aroused by the background story of either the victim or the transgressor, which might mean a lower consistency in the restorative orientation. Specifically, we expected that children's empathy for the victim would be conducive to a stronger treatment preference in the punitive orientation, whereas their empathy for the transgressor would be conducive to a stronger treatment preference in the restorative orientation.

The third research aim of the current study further addressed how young children endorse restorative treatment at the community level. We presented children with options involving community-wide engagement for treating the victim, the transgressor, and the community as a whole in the aftermath of a moral transgression. Except for the community-level treatments for the victim, which were all restorative treatments, the design of the community-level treatments for the transgressor and the community were differentiated into restorative treatments and retributive treatments. Furthermore, the consistency in children's restorative orientation with regard to community-level treatments for the transgressor and the community was assessed in light of the RR model. Young children's understanding of restorative justice along the dimension of community engagement is a hitherto unexplored area.

Finally, we were interested in exploring the developmental features of young children's understanding of restorative justice by observing the similarities and differences between the 5- and the 8-year-olds involved in the current study with regard to the above three research aims. Past research in early moral development has shown age differences in the understanding of distributive justice and procedural justice, where the age range of five to eight appears to be a critical period of change. In line with the knowledge that older children in early and middle childhood are more advanced in perspective taking (Selman, 1975; see Elfers et al., 2008), we predicted that 8-year-olds would have a higher level of understanding of restorative justice at the community level than their 5-year-old counterparts, which would be reflected by the higher consistency in the preference for a restorative orientation.

## Methods

### Participants

The participants were from a medium-sized city in Southwest China, including ninety-three 5- to 6-year-old children at a senior grade in a local public kindergarten (*M*_age_ = 5.67, *SD*_age_ = 0.34, 49.3% girls) and 100 second-graders in a local public primary school (*M*_age_ = 7.86, *SD*_*age*_ = 0.29, 46.0% girls). The kindergarten subsample and primary school subsample are referred to as 5- and 8-year-olds, respectively. To determine the sample size, we conducted power analysis using G^*^Power 3.1 and found that we would need 188 participants in total in our research design, so that the difference of young children's preference between restorative and retributive treatments could be detected with 80% power (two tails, α = 0.05, assuming small to medium effect size equals 0.3). Considering the possibility that some children might withdraw from the study, we recruited 193 children. It turned out that all recruited children agreed to participate at the beginning of the study and all of them completed the research process.

Consent forms were distributed and collected from the parents through an online platform before the implementation of the study. The children recruited were from middle-class families in the urban area. The educational level of the parents should be noted, as 6.2% had a graduate school education, and 45.6% had completed university education. A further 25.4% of the parents had finished vocational school, while 12.4% had completed high school. The percentage of parents with an education level of middle school or below was 5.2. Another 5.2% of parents did not provide information on their educational level.

The story type and background condition were the two between-subject factors. Participants were randomly assigned to four cells within each age group, resulting in the following distribution: 24 5- and 27 8-year-olds in the Stealing Story and transgressor background condition, 24 5- and 24 8-year-olds in the Stealing Story and victim background condition, 22 5- and 24 8-year-olds in the Harm-causing Story and transgressor background condition, and 23 5- and 25 8-year-olds in the Harm-causing Story and victim background condition.

### Materials

We undertook an interactive story reading and interview process to assess children's understanding of restorative justice in moral transgressions. The multidimensional meanings and abstract moral rules of restorative justice were embedded in the stories along with a series of questions that could be easily understood and answered by young children.

#### Story stimuli

The study used two colored picture books of A4 size depicting the following two prototypical moral transgressions that occurred in an animal community: property violation and physical harm. Children were randomly assigned to reading one of the storybooks. The Stealing Story was about a property violation (see online Supplementary Material), and the Harm-causing Story was about physical harm (see online Supplementary Material). The structure of the interview questions was the same for both stories.

#### Initial moral judgments

The children's responses to the act's acceptability were scored on a five-point scale with the following specifications: 1 (very wrong), 2 (wrong), 3 (neither wrong nor right), 4 (right), 5 (very right). In a similar vein, their responses to the actor's acceptability were scored on a 5-point scale with the following specification: 1 (very bad), 2 (bad), 3 (neither bad nor good), 4 (good), 5 (very good). In both cases, the scales were adapted from the rating scale used in the study of Smetana and Ball (2018).

#### Ranking of Restorative and Retributive Treatments

The experimenter informed the children that four treatments were proposed at an animal meeting and that they needed to rank the treatments from the most preferable to the least preferable using four red ballots of decreasing size. The children were further asked to explain their ranking. The first treatment was a psychological restorative solution suggesting that the transgressor should apologize to the victim. The second treatment was an actual restorative solution or an action-oriented solution, suggesting that the transgressor should restore the situation. In the case of the Stealing Story, it involved returning the property; in the case of the Harm causing Story, it involved helping the victim clean the farm. The third treatment was an actual retributive solution of imprisoning the transgressor. The fourth treatment was a psychological retributive solution involving criticizing the transgressor. The four treatments were presented to the children on one page of the storybook with a consistent format across participants. Some necessary explanation of the treatments was made to ensure that the children understood their meanings.

#### The Hardship Background and Second-Round Judgment

Next, the children were randomly assigned to read the hardship background of the transgressor or the victim. The hardship of the transgressor centered on its fate of being an orphan, and the hardship of the victim was also attributed to its fate of being an orphan, both of which led to the lack of socialization. Then, the experimenter repeated the questions regarding the act's and actor's acceptability and the ranking of the four treatments posed in the initial phase.

#### Community-Involved Judgments

The experimenter informed the children that the transgression had negative consequences on the community even though the transgressor conducted restorative action and apologized to the victim. Then, the experimenter turned to three sets of treatments that involved the participation of the community in the justice-seeking process in the future, namely, treatments for the victim, the transgressor, and the community as a whole. The experimenter asked the children to rank the options within the three sets of treatments from the most preferable to the least preferable using plastic stars of decreasing sizes. The options were presented in a consistent format across the participants. Whether they had a further explanation for their ranking was probed.

#### Set 1: Treatments for the Victim

(a) Help the victim recover from the harm or loss; (b) teach the victim how to protect itself or its property in the future; or (c) make friends with the victim to alleviate its sadness.

#### Set 2: Treatments for the Transgressor

(a) Expel the transgressor from the community; (b) educate the transgressor; (c) exclude the transgressor from group activities; and (d) let the transgressor serve the community. Options (a) and (c) are retributive treatments, while options (b) and (d) are restorative treatments.

#### Set 3: Treatments for the Community

(a) Expel the animal who misbehaved; (b) educate the community members (in the case of the Stealing Story, teaching each animal to protect its property; in the case of the Harm-causing Story, teaching each animal to keep the environment clean); (c) exclude the wrongdoer from group activities; and (d) ask every member of the community to shoulder the responsibility of helping the needy community members (with respect to the Stealing Story) or protecting the environment (with respect to the Harm-causing Story). While options (a) and (c) are retributive treatments, options (b) and (d) are restorative treatments.

### Procedure

Ethics approval for the current study was obtained from the Survey and Behavioral Research Ethics Committee of the Chinese University of Hong Kong. To test the feasibility of the materials, we conducted a pilot study at a kindergarten and a primary school in a medium-sized city in southwestern China. A total of eleven 5-year-olds (six boys and five girls) and ten 8-year-olds (five boys and five girls) participated in the pilot study. Generally, the instructions, stories, and questions were comprehensible to the children. Minor changes to the wording of the storybooks were made according to the children's feedback.

In the main study, participants were randomly assigned to one of the four conditions (i.e., two different types of stories across two different manipulations of hardship backgrounds). As in the case of the pilot study, an experimenter with doctoral training in developmental psychology conducted the main study. Subsequent to a short warm-up that involved playing Legos with the participants, the experimenter read the storybook and interviewed the children individually in a quiet room at their schools. At the end of each interview, the experimenter thanked the child and gave him or her a set of stationary as a small souvenir.

## Results

### Children's Ranking of Treatments at the Individual Level

Analyses of the data revealed that most children preferred restorative treatments to retributive treatments for the transgressor in both moral transgression situations. The results showed that 45.1 and 38.9% of the children ranked actual restoration as their first choice and second choice, respectively; 43.5 and 36.3% of the children ranked apology as their first choice and second choice, respectively; 64.8% of the children chose criticism as the third choice; and 85.0% of the children chose imprisonment as the least preferable choice. This pattern of results echoes our prediction related to the first research aim of the current study, which specified that young children were capable of showing preference for restorative treatments in unintentional moral transgressions. No gender difference was found in the ranking [apology: $\chi_{\left(\text{df}\;=\;3\right)}^{2}$ = 1.04, *p* = 0.791; restoration: $\chi_{\left(\text{df}\;=\;3\right)}^{2}$ = 0.51, *p* = 0.917; criticism: $\chi_{\left(\text{df}\;=\;3\right)}^{2}$ = 5.13, *p* = 0.163; jail: $\chi_{\left(\text{df}\;=\;3\right)}^{2}$ = 6.48, *p* = 0.091], and gender was not considered in further analyses. The percentage of each ranking (from 1st to 4th) of the four treatments, further differentiated into the percentage in the two age groups and the two types of stories concerning moral transgression, is reported in Table 1.

**Table 1:** Percentage of each ranking (1st-4th) for the four treatments.

		**Percentage of rankings for actual restoration (%)**			
		**1st**	**2nd**	**3rd**	**4th**
Age	5	40.9	39.8	15.1	4.3
	8	49.0	38.0	13.0	0.0
Story	Stealing	35.0	47.0	16.0	2.0
	Harm-causing	55.9	30.1	11.8	2.2
		**Percentage of rankings for apology (%)**			
		**1st**	**2nd**	**3rd**	**4th**
Age	5	39.8	30.1	15.1	15.1
	8	47.0	42.0	11.0	0.0
Story	Stealing	58.0	26.0	11.0	5.0
	Harm-causing	28.0	47.3	15.1	9.7
		**Percentage of rankings for imprisonment (%)**			
		**1st**	**2nd**	**3rd**	**4th**
Age	5	8.6	5.4	12.9	73.1
	8	0.0	0.0	4.0	96.0
Story	Stealing	3.0	1.0	8.0	88.0
	Harm-causing	5.4	4.3	8.6	81.7
		**Percentage of rankings for criticism (%)**			
		**1st**	**2nd**	**3rd**	**4th**
Age	5	10.8	24.7	57.0	7.5
	8	4.0	20.0	72.0	4.0
Story	Stealing	4.0	26.0	65.0	5.0
	Harm-causing	10.8	18.3	64.5	6.5

### The Effect of Age and Story Type on Individual-Level Treatment Ranking

As the children's rankings of the four treatments were ordinal data by nature, the following analysis used ordinal regression to test the effect of independent variables on the children's preference for the four treatments. We were interested in how age and story type and their interaction impacted the children's ranking of the treatments. Considering that the use of a continuous variable would be conducive to empty cells and lead to a violation of the parallel assumption that is required for ordinal regression (O'Connell, 2006), we treated children's age as a categorical predictor in the following ordinal regression analysis. Thus, age was dummy coded as 0 (5-year-olds) and 1 (8-year-olds). Story type was also dummy coded as 0 (Stealing Story) and 1 (Harm-causing Story). The interaction product of the two factors was created by multiplying dummy-coded age by dummy-coded story type. In four ordinal regression models, age, story type, and their interaction were predictors, whereas the children's ranking of actual restoration, apology, imprisonment, and criticism were the dependent variables.

The parameter estimates and model-fit outcomes of the ordinal regression models are shown in Table 2. Regarding actual restoration, the respective main effects of age and story type on its ranking were not significant (*logit*_age_ = −0.67, *odds ratio* = 0.51, *p* = 0.078, 95% CI[−1.41, 0.08]; *logit*_story_ = −0.49, *odds ratio* = 0.61, *p* = 0.210, 95% CI[−1.26, 0.28]), but the interaction effect of the two variables was significant (*logit*_interation_ = 2.41, *odds ratio* = 11.11, *p* < 0.001, 95% CI[1.27, 3.55]), as was the overall model fit (see Table 2). Further analysis revealed that for the Stealing Story, the children's age was not significantly associated with their ranking of actual restoration (*logit* = −0.71, *odds ratio* = 0.49, *p* = 0.064, 95% CI[−1.47, 0.04]). However, for the Harm-causing Story, the children's age was significantly associated with their ranking of actual restoration (*logit* = 1.65, *odds ratio* = 5.22, *p* < 0.001, 95% CI[0.79, 2.51]), which indicated that the older children were more likely to give a higher ranking to actual restoration than younger children in the context of the Harm-causing Story. The children's ranking of apology was significantly predicted by age (*logit*_age_ = 1.23, *odds ratio* = 3.41, *p* = 0.002, 95% CI[0.44, 2.02]), which indicated that older children gave more credit to psychological restoration; but the ranking of apology was not predicted by story type and the interaction (*logit*_story_ = −0.58, *odds ratio* = 0.56, *p* = 0.133, 95% CI[−0.13, 0.18]; *logit*_interaction_ = −0.96, *odds ratio* = 0.38, *p* = 0.082, 95% CI[−2.05, 0.12]), Regarding the ranking of imprisonment, the effect of age was significant (*logit*_age_ = −1.90, *odds ratio* = 0.15, *p* = 0.019, 95% CI[−3.48, −0.32]), while the effect of story type and the interaction was not significant (*logit*_story_ = 0.70, *odds ratio* = 2.01, *p* = 0.136, 95% CI[−0.22, 1.61]; *logit*_interaction_ = −0.62, *odds ratio* = 0.54, *p* = 0.586, 95% CI[−2.83, 1.60]). These results indicated that older children gave less priority to imprisonment as a way to address moral transgression. Regarding the ranking of criticism, the respective effects of age (*logit*_age_ = −0.02, *odds ratio* = 0.98, *p* = 0.960, 95% CI[−0.83, 0.79]), story type (*logit*_story_ = 0.44, *p* = 0.288, *odds ratio* = 1.56, 95% CI[−0.37, 1.26]), and their interaction (*logit*_interaction_ = −0.82, *odds ratio* = 0.44, *p* = 0.165, 95% CI[−1.99, 0.34]) were all found to be non-significant.

**Table 2:** Results of ordinal regression analysis with the ranking of actual restoration, apology, imprisonment, and criticism as respective outcome variables.

**Outcome variable**	**Predictors**	**Logistic coefficient (*p-*value)**	**Odds ratio^**a**^**	**Model fitting **χ^2^** (*df* = 3) (*p*-value)**	**Pseudo *R*^**2**^ (Nagelkerke)**
Actual restoration	Age	−0.67 (0.078)	0.51	26.66 (<0.001)	0.15
	Story	−0.49 (0.210)	0.61		
	Age*Story	2.41 (<0.001)	11.11		
Apology	Age	1.23 (0.002)	3.41	24.16 (<0.001)	0.13
	Story	−0.58 (0.133)	0.56		
	Age*Story	−0.96 (0.082)	0.38		
Imprisonment	Age	−1.90 (0.019)	0.15	24.64 (<0.001)	0.18
	Story	0.70 (0.136)	2.01		
	Age*Story	−0.62 (0.586)	0.54		
Criticism	Age	−0.02 (0.960)	0.98	3.76 (0.288)	0.02
	Story	0.44 (0.288)	1.56		
	Age*Story	−0.82 (0.165)	0.44		

a *5-year-olds and the Stealing Story group served as the reference groups*.

### Correlation Analysis of Act and Actor Acceptability and Treatment Ranking

To examine whether the children's ranking of the treatments was correlated with their judgment of act and actor acceptability, we computed the correlation between the children's ratings of acceptability and their treatment ranking using Spearman's rho test (see Table 3). The results showed that the worse the act was judged by the children, the stronger their preference was for actual restoration (ρ = −0.17, *p* = 0.017) and the lower their preference for apology (ρ = 0.18, *p* = 0.011). Regarding actor acceptability, the correlation analysis indicated that the worse the actor was judged by the children, the higher they ranked imprisonment (ρ = −0.20, *p* = 0.005) and criticism (ρ = −0.20, *p* = 0.005) and the lower they ranked apology (ρ = 0.23, *p* = 0.001). These results revealed that the children's preference for retributive treatment was correlated with their judgment of actor acceptability rather than act acceptability.

**Table 3:** Correlations of acceptability and preference for the four treatments.

	**Actual restoration**	**Apology**	**Imprisonment**	**Criticism**
Act acceptability	−0.17 (*p* = 0.017)	0.18 (*p* = 0.011)	−0.07 (*p* = 0.324)	0.07 (*p* = 0.370)
Actor acceptability	0.08 (*p* = 0.290)	0.23 (*p* = 0.001)	−0.20 (*p* = 0.005)	−0.20 (*p* = 0.005)

*Correlations were computed by Spearman's rho value. Act acceptability and actor acceptability were scored on 5-point scales with 5 = very right and very good, respectively; the four treatments were rated on a scale ranging from 1 = the least preferable choice to 4 = the most preferable choice*.

### The Effects of Transgressor and Victim Hardship on Treatment Ranking

To examine whether knowing the hardship background of the transgressor or the victim would lead the children to change their ranking of the four treatments, we compared children's pre- and post-ranking of the treatments using the Wilcoxon signed-rank test. The results of the between-subject comparisons are listed in Table 4.

**Table 4:** Comparisons of children's pre- and post-preference for moral treatments.

	**Transgressor hardship**				**Victim hardship**			
	**Negative rank^**a**^**	**Positive rank^**b**^**	**Ties**	***Z* (*p*-value)**	**Negative rank**	**Positive rank**	**Ties**	***Z* (*p*-value)**
Res_post_-Res_pre_^c^	35	22	39	−1.71 (0.088)	23	27	47	−0.26 (0.797)
Apo_post_-Apo_pre_^c^	18	38	40	−2.36 (0.018)	37	17	43	−3.07 (0.002)
Imp_post_-Imp_pre_^c^	10	7	79	−0.03 (0.980)	2	15	80	−3.18 (0.001)
Cri_post_-Cri_pre_^c^	24	18	54	−1.23 (0.218)	15	21	61	−1.03 (0.304)

a *The number of pairs for which the post-ranking was lower than the pre-ranking*.

b *The number of pairs for which the post-ranking was higher than the pre-ranking*.

c *Res_pre_ and Res_post_ refer to the children's pre- and post-ranking of actual restoration, respectively; Apo_pre_ and Apo_post_ refer to the children's pre- and post-ranking of apology, respectively; Imp_pre_ and Imp_post_ refer to the children's pre- and post-ranking of imprisonment, respectively; and Cri_pre_ and Cri_post_ refer to the children's pre- and post-ranking of criticism, respectively*.

The results indicated that the ranking of apology as a solution was higher after than it was before the children learned about the hardship of the transgressor (*Z* = −2.36, *p* = 0.018). However, after hearing the victim's hardship story, the children ranked apology as a less preferable solution (*Z* = −3.07, *p* = 0.002) and believed that a harsher punishment of putting the transgressor into prison was a more appropriate solution (*Z* = −3.18, *p* = 0.001). Other comparisons between the pre- and post-rankings were not significant (see Table 4). These findings suggested that the hardship of the involved parties had an impact on the priority given to psychological restoration and actual punishment by the children: In the case when they were told that the transgressor had experienced hardship, they were more likely to support apology as a solution; if they were told that the victim had experienced hardship, they were more likely to support putting the transgressor into prison and less likely to support accepting an apology from the transgressor. These results echo our prediction related to the second research aim of the current study, which specified that children's consistency in their restorative vs. punitive orientation would be affected by their empathy for the victim or the transgressor.

### Children's Preference for Community-Involved Treatments

In the last part of the storybook, we presented children with a series of solutions at the community level focusing separately on the victim, the transgressor, and the community. We were interested in examining the ways in which the children's rankings at the community level were associated with age, story type, and the background of the involved parties. Along with listing the percentages associated with the children's rankings of the solutions that addressed the victim, transgressor, and community, Table 5 shows the results of ordinal regression. Each solution's ranking appears as the outcome variable, and age (0 = 5-year-olds, 1 = 8-year-olds), story type (0 = Stealing Story, 1 = Harm-causing Story) and hardship background (0 = transgressor background, 1 = victim background) are the predictors.

**Table 5:** Percentage of ranking for community-level solutions and the results of ordinal regressions predicting rankings from age, story type, and hardship background.

		**Percentage of ranking (%)**			**Logits**^**a**^ **(*****p*** **value)**		
		**1st**	**2nd**	**3rd**	**Age**	**Story**	**Background**
Treatments	Helping	49.2	24.4	25.9	−0.45	−0.28	0.76
for the victim					(0.108)	(0.308)	(0.006)
	Teaching	25.4	44.0	30.1	0.07	−0.07	−0.82
					(0.800)	(0.808)	(0.003)
	Comforting	25.4	31.1	43.0	0.27	−0.20	−0.05
					(0.315)	(0.448)	(0.842)
Treatments for	Expelling	3.6	4.1	6.2	–	0.47	0.37
the transgressor					–	(0.295)	(0.412)
	Excluding	1.6	6.2	69.9	0.37	0.50	0.80
					(0.237)	(0.116)	(0.013)
	Educating	70.5	24.9	2.1	1.04	−0.28	−0.73
					(0.002)	(0.392)	(0.026)
	Serving	24.4	62.7	8.3	–	−0.12	0.23
					–	(0.677)	(0.430)
Treatments for	Expelling	8.8	8.8	6.2	−2.60	−0.34	0.83
the community					(<0.001)	(0.358)	(0.026)
	Excluding	3.6	13.5	62.2	−0.53	0.13	0.31
					(0.069)	(0.645)	(0.282)
	Educating	39.9	44.6	6.7	0.42	0.68	0.24
					(0.123)	(0.014)	(0.383)
	Responsibility-taking	48.7	31.6	14.0	1.48	−0.67	−0.48
					(<0.001)	(0.018)	(0.086)

*For the logits of age, story type, and background, the reference groups are 5-year-olds, the Stealing Story group, and the transgressor background group, respectively*.

Table 5 indicates that compared to the children who knew the hardship story of the transgressor, those who knew the hardship background of the victim were more likely to prioritize helping the victim recover from the harm or restore the loss (*logit* = 0.76, *odds ratio* = 2.13, *p* = 0.006, 95% CI[0.21, 1.30]) but less likely to teach the victim to protect itself or its property (*logit* = −0.82, *odds ratio* = 0.44, *p* = 0.003, 95% CI[−1.36, −0.28]).

With regard to the treatment of the transgressor, the older children were more likely to favor educating the transgressor (*logit* = 1.04, *odds ratio* = 2.83, *p* = 0.002, 95% CI[0.39, 1.69]) when they were informed of its hardship than the group that received the information about the hardship of the victim. In addition, compared to the children who knew the hardship of the transgressor, the children who knew the hardship of the victim were more inclined to exclude the transgressor from group activities (*logit* = 0.80, *odds ratio* = 2.22, *p* = 0.013, 95% CI[0.17, 1.43]) and were less likely to give support to educating the transgressor (*logit* = −0.73, *odds ratio* = 0.48, *p* = 0.026, 95% CI[−1.38, −0.09]).

Concerning the solution for the whole community, the results showed that the older children were more likely to give priority to the solution requiring the community members to share the responsibility (*logit* = 1.48, *odds ratio* = 4.39, *p* < 0.001, 95% CI[0.91, 2.05]) and were less willing to support the solution that suggested expelling the transgressor from the community (*logit* = −2.60, *odds ratio* = 0.07, *p* < 0.001, 95% CI[−3.54, −1.66]). Compared to the Stealing Story group, the Harm-causing Story group was more likely to favor the solution that suggested educating community members to prevent future transgression (*logit* = 0.68, *odds ratio* = 1.97, *p* = 0.014, 95% CI[0.14, 1.22]) but less likely to ask each community member to shoulder the responsibility (*logit* = −0.67, *odds ratio* = 0.51, *p* = 0.018, 95% CI[−1.23, −0.12]). In addition, compared with the children who knew the hardship background of the transgressor, the children who knew the hardship background of the victim were more likely to support the expulsion of future transgressors from the community (*logit* = 0.83, *odds ratio* = 2.30, *p* = 0.026, 95% CI[0.10, 1.57]). The pattern of results reported in this subsection addressed the third research aim of the current study in uncovering young children's understanding of restorative justice at the community level, which fills a knowledge gap in the field.

### The Consistency of the Children's Restorative Orientation

In our current study, the consistency of the children's restorative orientation refers to their consistent preference for restorative treatments across different situations within each story of moral transgression. First, we were interested in the consistency of the children's preference for restorative justice before and after learning about the hardship background of the victim or the transgressor at the individual level. The treatments were ranked from one to four, and there were two restorative treatments and two retributive treatments. In the case that a child's pre- and post-rankings for the two restorative treatments were always at or above the second-ranking (i.e., first or second), he or she was considered to be consistently supportive of restorative treatments.

Regarding the community-level treatments, the treatments for the transgressor and the community each included two restorative treatments and two retributive treatments. The children who top-ranked the two restorative treatments for the transgressor and top-ranked the two restorative treatments for the community were regarded as having a consistent restorative orientation. Table 6 shows the number of participants with a consistent or inconsistent restorative orientation at both the individual and community levels. Those participants who showed a consistent retributive orientation were excluded from the analysis.

**Table 6:** Number of children with a consistent or an inconsistent restorative orientation.

	**Individual level**		**Community level**		**Both levels**	
**Age**	**Incons**.	**Cons**.	**Incons**.	**Cons**.	**Incons**.	**Cons**.
5	45	44	47	43	66	24
8	46	54	16	84	56	44
χ^2^ (*df* = 1)	0.39 (*p* = 0.315)		28.04 (*p* < 0.001)		6.19 (*p* = 0.013)	

*Incons., inconsistent; cons., consistent. Children who had a consistent retributive orientation were excluded from the analyses (four 5-year-olds at the individual level, three 5-year-olds at the community level, and three 5-year-olds at both levels)*.

As shown in Table 6, there was no age difference in the number of children with consistent and inconsistent restorative orientations at the individual level [$\chi_{\left(\text{df}\;=\;1\right)}^{2}$ = 0.39, *p* = 0.531]. However, at the community level, there were significantly more 8-year-olds who chose the restorative treatments consistently than 5-year-olds [$\chi_{\left(\text{df}\;=\;1\right)}^{2}$ = 28.04, *p* < 0.001]. When combining the children's choices at the individual level and the community level, significantly more 8-year-olds had a consistent restorative orientation than 5-year-olds [$\chi_{\left(\text{df}\;=\;1\right)}^{2}$ = 6.19, *p* = 0.013]. In other words, when considering treatments that involved the transgressor and the victim only at the individual level, there was a similar proportion of 5- and 8-year-olds who held a consistent restorative orientation before and after learning about the hardship story of one of the involved parties. In contrast, when considering treatments involving the engagement of the whole community, more 8- than 5-year-olds held a consistent restorative orientation. This finding echoes our prediction made on the age differences in the understanding of restorative justice, based on the development of perspective-taking during early and middle childhood.

## Discussion

The current study systematically investigated children's understanding of the multidimensional meanings of restorative justice. We embedded a choice-based paradigm into newly developed story materials to examine children's preference for restorative or retributive treatments. The results revealed that most 5- and 8-year-olds demonstrated a sense of restorative justice in response to unintentional moral transgressions. Specifically, children preferred restorative treatments to retributive treatments in both property violation and harm-causing scenarios, and their endorsement of apology, a type of psychological restoration, developed with age. We also observed that the children's preference for restorative treatments was associated with their judgment of the acceptability of the moral transgression and could be influenced by knowledge of the hardship background of the victim or transgressor. In addition, the children also extended their preference for restorative treatment to community-level engagement.

### Knowledge Advancement Centered on the Three Critical Differentiations of the Treatments for Moral Transgressions

A central aim of the current study was to examine the endorsement of restorative treatments in young children. The study addressed this aim by applying three critical treatment differentiations in our materials to gain deeper insights into children's understanding of restorative justice. The first differentiation involves children's preference for restorative options vs. retributive options. Restorative treatments, including both apology and actual restitution, are important for maintaining social harmony and repairing relationships and trust (Drell and Jaswal, 2016; Ma et al., 2018). Our findings indicated that most children in our sample, including those as young as 5 years of age, showed a preference for both forms of restoration. Notably, these findings, elicited by a choice-based paradigm, concur with the restoration orientation found in other pioneering studies using an action-based paradigm (Riedl et al., 2015; Yang et al., 2021). This implied that a sense of restorative justice could be fostered in young children's minds.

The second differentiation that we made was between the psychological treatments and actual or behavioral treatments within the restorative and retributive orientations. Our findings showed that most children preferred actual restoration as the first choice and psychological restoration as the second choice. The more severe the transgression was judged to be, the more endorsement was given to actual restoration, which required the transgressor to provide actual compensation to the victim. Previous studies found no age differences in children's judgment regarding whether an offender was deserving of punishment for certain moral transgressions (Smetana, 1981). However, when we differentiated punishment into psychological and actual punishment, we found that the endorsement of actual punishment decreased with age, although the endorsement of psychological punishment did not change. Given that the moral transgressions described in both stories were unintentional, the decreased endorsement of punishment in older children might be attributable to their developing theory of mind, which enables them to better understand other's mental states, intentions, emotions, and thoughts (see Flavell, 1999). The results also echo Piaget's observation that older children have a reduced preference for punishment as they develop from the egocentric stage to the cooperation stage (Piaget, 1932/1997).

The third differentiation was between restorative treatments at the individual level and the community level. The individual-level treatments focused on only the two parties—the transgressor and the victim. Previous studies on punishment and apology have seldom taken the well-being of the community into consideration (e.g., Cushman, 2008; Oostenbroek and Vaish, 2019). Community-level treatments consider the community as a whole in addressing wrongdoing. Piaget (1932/1997) posited that exclusion causes the offender to feel isolated and thus motivates him or her to return to normal social relations. Previous studies have found that children accepted the social exclusion of in-group members out of consideration for group norms and group functioning (Killen et al., 2013; Hitti et al., 2014). The current study suggested that even when the community is harmed by a transgression, many young children are reluctant to exclude an unintentional transgressor and overwhelmingly endorse restorative options, including educating or responsibility taking. The findings also implied that young children opt for social support in addressing wrongdoings instead of using harsh measures of societal deterrence.

### Consistency of the Restorative Orientation

Through the lens of the RR model, the consistency analysis in the preference for restorative treatments revealed that a substantial portion of 5- and 8-year-olds had reached the initial level of explicit understanding of restorative justice. Children who indicated certain restorative preferences but did not demonstrate consistency might be perceived as having an understanding of restorative justice at the implicit level. Although the children were asked to justify their treatment preference for moral transgressions in the study, articulated explanations of restorative justice were not witnessed. The most typical answer for a justification of their treatment preference was “I don't know.” In the remaining cases, the children somewhat restated their preferences in their own words or talked about unrelated issues. Thus, regarding the participants' understanding of restorative justice, no evidence was found to support their general attainment of Level E2/3, the high level of explicit understanding postulated in the RR model, at which representations are conscious and verbally articulable. It was evident that the older children showed higher consistency in their restorative orientation at the community level, reflecting a more comprehensive mastery of the multidimensional concept of restorative justice. No significant difference was found between the 5- and the 8-year-olds in their restorative orientation consistency at the individual level. Consistency in choosing restorative treatments for the transgressor in this case required a mature and consolidated mastery of the concept of restorative justice despite hearing the hardship story of the victim. This might explain why even the 8-year-olds did not show higher consistency than their 5-year-old counterparts with respect to the restorative orientation. As explicated by Karmiloff-Smith (1992/1999), the RR model postulates the development of different levels of conceptual understanding along the implicit-explicit continuum and is not an aged-based theoretical framework. Nonetheless, with respect to young children's understanding of a certain concept, such as restorative justice, along this continuum, the RR model can also serve as a lens to discern the developmental characteristics of different age groups.

### Limitations and Future Directions

Despite the interesting findings of the current study, it has several limitations. First, the participants in the current study were all from families of middle socioeconomic status with relatively good educational backgrounds living in a medium-sized city in Southwest China. Thus, it might not be possible to generalize the results to explain the restorative orientation of children from other cultural and socioeconomic backgrounds. In the future, examining the preference for restorative treatments of children from different backgrounds in cross-cultural settings would be worthy of our endeavors. Cultural differences in moral development during early and middle childhood have been found in empirical studies (see, for instance, Lau et al., 2012; Chiu Loke et al., 2014; Paulus, 2015). As the current study on young children's understanding of restorative justice is pioneering and involves only a Chinese sample, we are not yet in a position to contribute to the discourse on the issue of universality vs. cultural specificity in early moral development. Considering that the understanding and practice of restorative justice is a global concern, cross-cultural research in this topic area constitutes a valuable future direction. Second, we acknowledge that both of the moral transgression scenarios designed in the current study were unintentional by nature. Future research can explore how children endorse restorative treatments in the context of intentional transgression and how children's restorative preferences vary across different levels of transgression severity. Preference in the practice of restorative justice vs. retributive justice is a complex jurisprudence issue that is value-laden and depends on the nature of the transgression and its contexts. In the current study, we found that children's preference for retributive treatment was negatively and significantly correlated with actor acceptability. Investigation into children's restorative vs. punitive orientation in the context of intentional moral transgressions would be an interesting focus for future studies. Finally, in employing a cross-sectional design in the current study, we were not able to rule out the possibility that the differences between the two age groups were due to the birth cohort effect induced by shared temporal experiences. In the future, the application of a rigorous longitudinal design is recommended to verify the developmental characteristics of the understanding of restorative justice.

## Conclusions

Notwithstanding the limitations of the current study, it has important theoretical, methodological, and practical implications. Interpreting our data through the lens of the RR model, we attained a more refined perception of young children's levels of understanding regarding restorative justice. The study also serves to extend the RR model's scope of application from cognitive development (e.g., mathematical cognition, language learning) to social cognition. On the methodological plane, the interactive storybooks on moral transgressions, in their current or further modified form, can serve as a new research tool for eliciting children's understanding of restorative justice vs. retributive justice. On the practical plane, the findings of the current study can provide insights for parents and teachers with regard to the cultivation of restorative justice in young children. Such an early cultivation is the most desirable way to facilitate a good understanding of the moral concept among citizens, representing the cornerstone of the successful practice of restorative justice vs. retributive justice in any society. It is encouraging to find that children as young as 5 years of age in this sample showed an understanding of restorative justice, admittedly a sophisticated concept. The finding that young children have different levels of understanding of this moral concept calls for further deliberation of how different educational means can be applied to children to enhance their acquisition and mastery of this concept. It is recommended to promote interactive reading activities related to restorative justice in family and kindergarten settings. Small group discussions regarding a restorative orientation vs. a retributive orientation can be arranged for primary school students using moral transgression scenarios. To facilitate young children's understanding of restorative justice at the implicit and explicit levels, intervention programs can be further designed to examine the role of exposure to stories of restorative justice and the role of various types of dialogues in the restorative orientation.

## Data Availability Statement

The datasets presented in this article are not readily available because authors have not asked for parents' permission to make public the interview record of the young children, we are not in the position to make such raw data available. Requests to access the datasets should be directed to Zheng Zhou, zhouzheng@swufe.edu.cn.

## Ethics Statement

The studies involving human participants were reviewed and approved by Survey and Behavioral Research Ethics Committee of the Chinese University of Hong Kong. Written informed consent to participate in this study was provided by the participants' legal guardian/next of kin.

## Author Contributions

Both ZZ and WW contributed to the conception and design of the study. ZZ conducted the experiment and analyzed the data. Both authors engaged in the interpretation and discussion of the findings. WW reviewed and revised the first draft of the manuscript written by ZZ. Both authors contributed to the final revision and approved the submitted version subsequent to a close reading.

## Funding

The data collection of the present study was supported the Department of Educational Psychology at the Chinese University of Hong Kong. The publication fee of this manuscript is supported by The Research Funds for the Young Scholars, Southwestern University of Finance and Economics (Grant Number: 230600001002020019).

## Conflict of Interest

The authors declare that the research was conducted in the absence of any commercial or financial relationships that could be construed as a potential conflict of interest.

## Publisher's Note

All claims expressed in this article are solely those of the authors and do not necessarily represent those of their affiliated organizations, or those of the publisher, the editors and the reviewers. Any product that may be evaluated in this article, or claim that may be made by its manufacturer, is not guaranteed or endorsed by the publisher.
