# Supplementary material for: Young Children's Understanding of Restorative Justice
Source: Front Psychol. 2021 Sep 28;12:715279. doi: 10.3389/fpsyg.2021.715279 (PMC8506036; doi:10.3389/fpsyg.2021.715279)

The Stealing Story

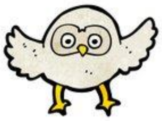
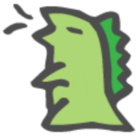
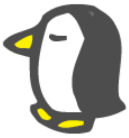
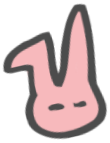
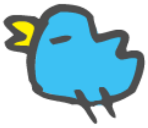

小象和小猫头鹰的故事

A story of an elephant and an owl

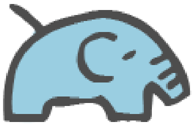
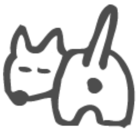
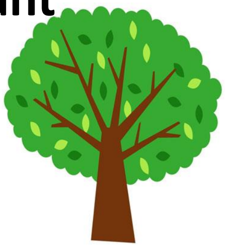

1

小象在森林里种了一棵苹果树，每天辛勤地浇灌它，苹果树一天天长大，结了5个大大的红苹果。

A small elephant planted an apple tree in the forest, watered the tree every day, and grew five apples on the tree.

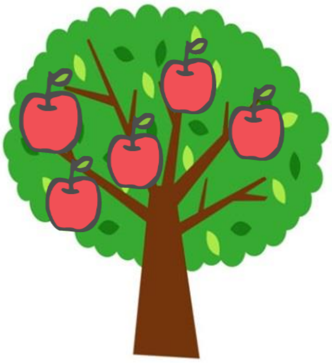
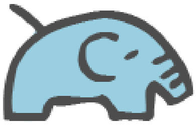

2

一天一大早，小象来到苹果树下，发现他的苹果有几个不见了！

One day, in the morning, when the elephant came to the tree, it found that some apples were missing!

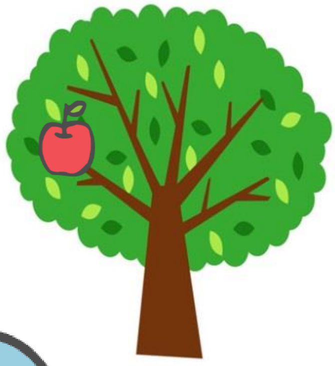

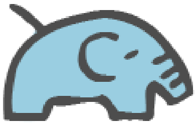

3

小象告诉了森林里的小动物，小动物们都不知道为什么苹果不见了，大家都很惊慌，说：“太可怕了，看来我们农场来了小偷了！”

The elephant told this to the other animals in the forest, and they all had no idea why the apples were missing. Everyone was anxious and said, “It’s horrible; it seems that a thief has come to our forest!”

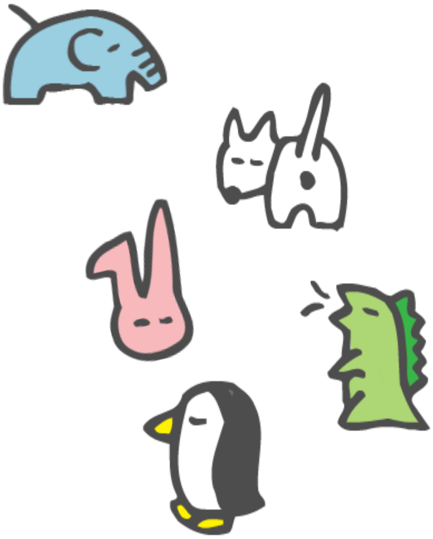

4

第二天，小象来到苹果树下，发现小猫头鹰正叼走它的苹果。小象生气地说：“原来是你，偷走了我的苹果！”小猫头鹰却说道：“我不知道这是你的呀。”

The next day, when the elephant came to the tree, it found that an owl was taking away an apple! The elephant said angrily, “It’s you who stole my apples!” The owl said, “I didn’t know that they were yours.”

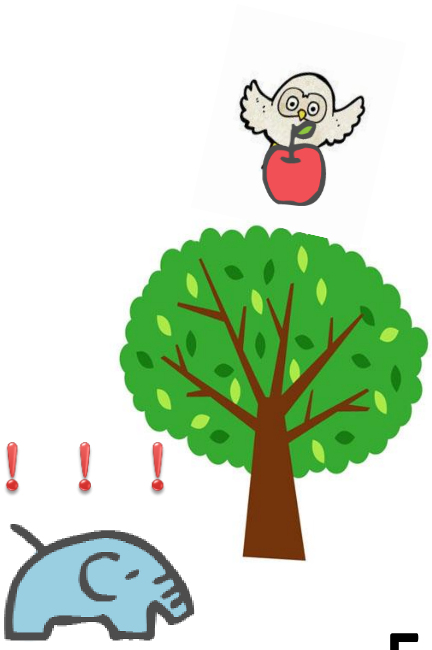

5

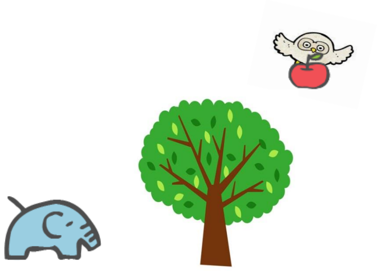

- 1. 小猫头鹰拿走小象的苹果对吗？ Was it right or wrong for the owl to take away the apple?
- Responses were scored on a 5-point scale ranging from 1 (very wrong) to 5 (very right).

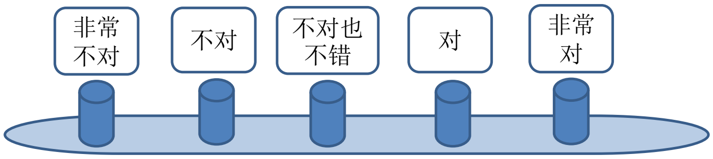

6

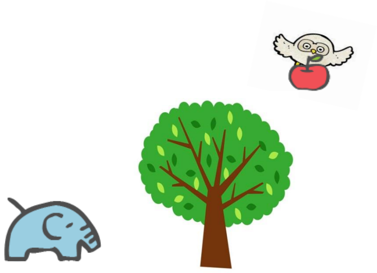

- 2. 小猫头鹰是好还是坏呢？Is the owl good or bad? Responses were scored on a 5-point scale ranging from 1 (very bad) to 5 (very good).

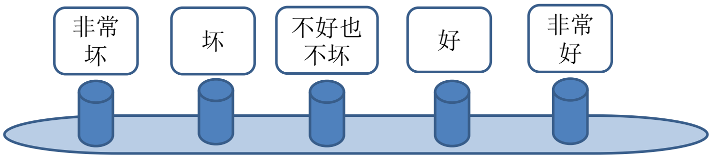

7

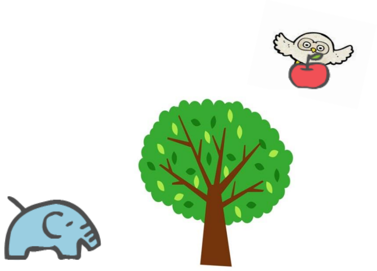

- 3. 现在应该怎么办呢？
- What should be done now?

8

召开森林大会 Forest Meeting

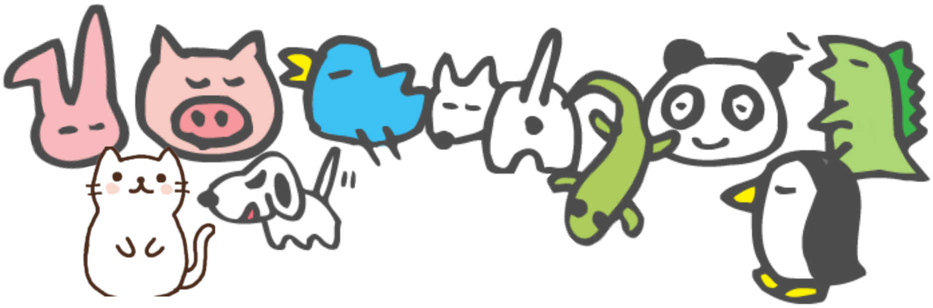

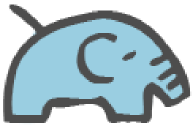

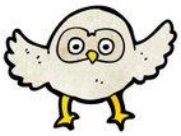

9

1. 小兔说：
A rabbit said,

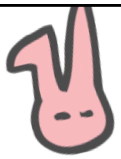

- “应该让小猫头鹰给小象道歉。”
- “The owl should apologize to the elephant.”

现在森林大会上四个小动物提出了这四个观点，我们要经过大家投票来决定哪一个最好。
Four animals proposed four views, we needed to vote to decide which one was the best.

2. 小猫说：
A cat said,

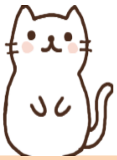

- “应该让小猫头鹰种苹果还给小象。”
- “The owl should plant apples and return them to the elephant.”

3. 小狗说：
A dog said,

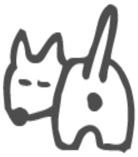

- “应该把小猫头鹰关到笼子里。”
- “The owl should be locked in a cage.”

4. 小鸟说：
A bird said,

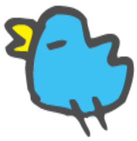

- “应该批评小猫头鹰。”
- “The owl should be criticized.”

10

投给小猫
Vote for the cat

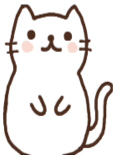

- “应该让小猫头鹰种苹果还给小象。”
- “The owl should plant apples and return them to the elephant.”

小猫头鹰应该还多少苹果给小象呢？How many apples should the owl give back to the elephant?

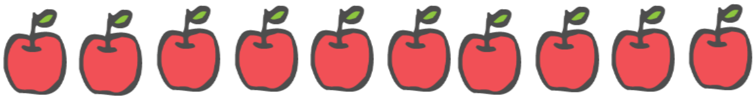

11

投给小狗
Vote for the dog

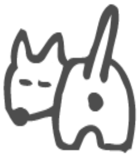

- “应该把小猫头鹰关到笼子里。”
- “The owl should be locked in a cage.”

应该把小猫头鹰关多少天呢？

How many days should the owl be locked in the cage?

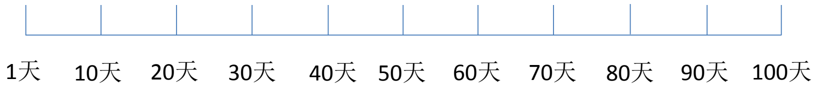

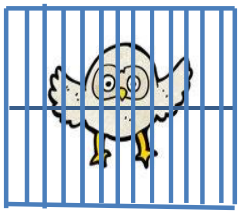

12

(Victim’s background)

- 森林大会上，大家发现，小象没有爸爸妈妈，它只能靠自己种苹果树来填饱肚子。现在，小猫头鹰拿走了小象的苹果，小象就没有吃的了。

- In the forest meeting, we learnt that the elephant is an orphan, and it could only plant the apple tree to provide food for itself. Now, the elephant has nothing to eat because the owl took away its apples.

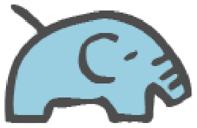

13

(Transgressor’s background)

- 森林大会上，大家发现，小猫头鹰原来没有爸爸妈妈，没有人教它怎么去捕食，所以当小猫头鹰看到树上有苹果，就去摘了苹果当食物。

- In the forest meeting, we learnt that the owl is an orphan, and no one has ever taught it how to forage for food. Thus, when the owl saw apples on the tree, it took them away for food.

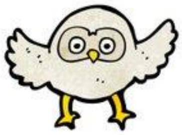

14

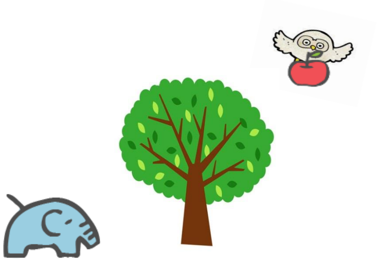

- 1. 你现在知道了小象（或者小猫头鹰）的故事，小猫头鹰拿走小象的苹果对吗？Now that you know the story of the elephant (or the owl), was it right or wrong for the owl to take away the apples?
- Responses were scored on a 5-point scale ranging from 1 (very wrong) to 5 (very right).

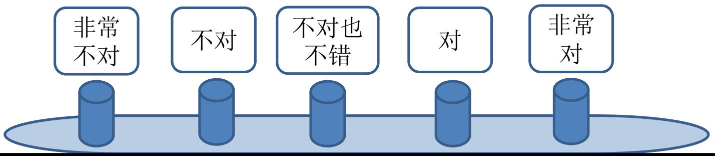

15

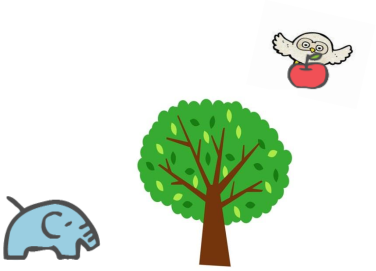

- 2. 小猫头鹰是好还是坏呢？Is the owl good or bad? Responses were scored on a 5-point scale ranging from 1 (very bad) to 5 (very good).

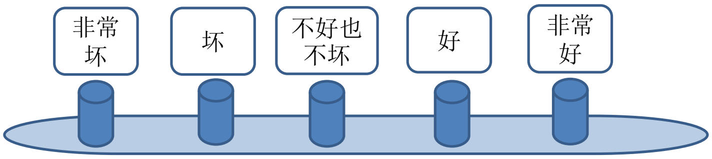

16

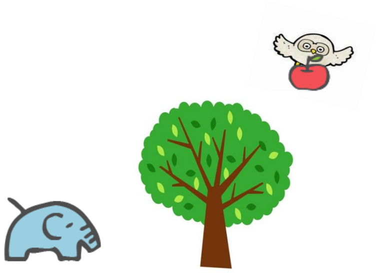

- 3. 现在应该怎么办呢？
- What should be done now?

17

继续召开森林大会

Forest Meeting continued…

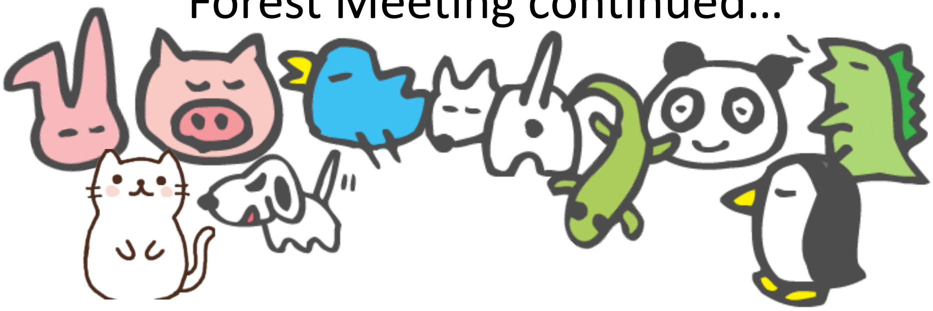

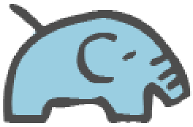

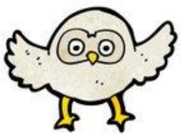

18

1. 小兔说：
A rabbit said,

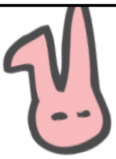

- “应该让小猫头鹰给小象道歉。”
- “The owl should apologize to the elephant.”

现在森林大会上四个小动物提出了这四个观点，我们要经过大家投票来决定哪一个最好。
Four animals proposed four views, we needed to vote to decide which one was the best.

2. 小猫说：
A cat said,

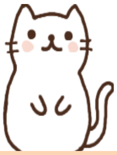

- “应该让小猫头鹰种苹果还给小象。”
- “The owl should plant apples and return them to the elephant.”

3. 小狗说：
A dog said,

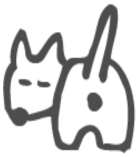

- “应该把小猫头鹰关到笼子里。”
- “The owl should be locked in a cage.”

4. 小鸟说：
A bird said,

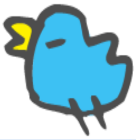

- “应该批评小猫头鹰。”
- “The owl should be criticized.”

19

投给小猫
Vote for the cat

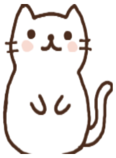

- “应该让小猫头鹰种苹果还给小象。”
- “The owl should plant apples and return them to the elephant.”

小猫头鹰应该还多少苹果给小象呢？
How many apples should the owl give back to the elephant?

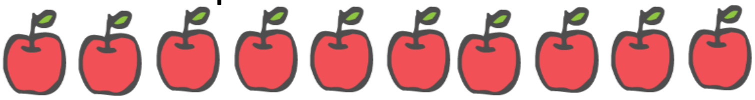

20

投给小狗

Vote for the dog

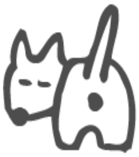

- “应该把小猫头鹰关到笼子里。”
- “The owl should be locked in a cage.”

应该把小猫头鹰关多少天呢？

How many days should the owl be locked in the cage?

1天 10天 20天 30天 40天 50天 60天 70天 80天 90天 100天

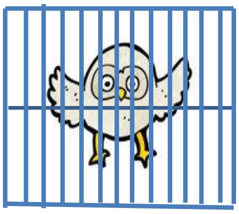

21

问题
Questions

- 小猫头鹰的行为会影响到整个森林吗？
- Does the owl’s behavior impact the whole forest?

会 Yes　　不会 No

22

- 如果你选会的话，请问小猫头鹰的行为对森林的影响程度有多大呢？How much does the owl’s behavior impact the forest？Responses were scored on a 4-point scale ranging from 1 (no impact) to 4 (very strong impact).

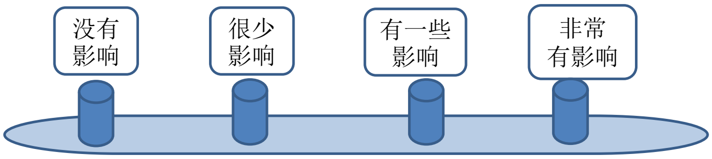

23

以前的森林是一个欢乐的大家庭，自从小猫头鹰偷走小象的苹果之后，森林里其他动物们都非常紧张，每人都担心自己的东西被偷走。虽然后来小猫头鹰还了5个苹果给小象，并向小象道了歉，这里已经不再是以前的欢乐森林了。

The forest used to be a happy family. Since the owl stole the apples, the animals have become anxious. Everyone worries that their own possessions will also be stolen. Although the owl has returned five apples and apologized to the elephant, the forest is no longer a happy forest as before.

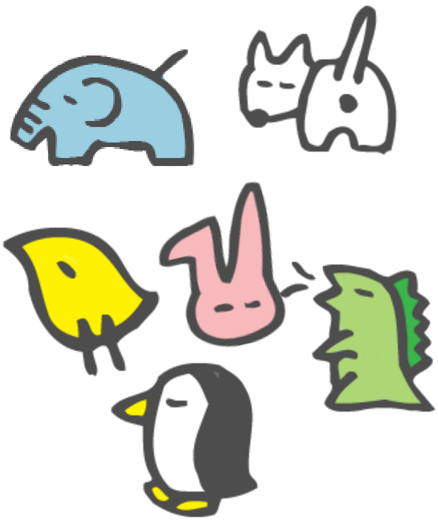

24

在这件事之后，作为森林的一员，我们应该怎么对待小象呢？**As members of the forest, how should we treat the elephant after this case? Please rank the options from the best to the worst.**

帮它种树，让他以后还可以继续吃苹果
help the elephant plant an apple tree

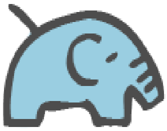

教它怎样保护自己的苹果树
teach the elephant how to protect the apple tree

和它做朋友，让它不要太难过
make friends with the elephant

25

在这件事之后，作为森林的一员，我们应该怎么对待小猫头鹰呢？**As members of the forest, how should we treat the owl after this case? Please rank the options from the best to the worst.**

把它赶出森林 expel it from the forest

教会它正确的寻找食物的办法
teach it the appropriate way to find food

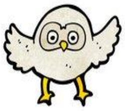

不让它和大家一起玩游戏
do not allow it to join any game with other animals

让它夜里做森林的保安，保护森林安全
let it serve as a guard in the forest

26

在这件事之后，作为森林的一员，我们社群应该做出什么改变呢？
As members of the forest, what changes should we make following this incident? Please rank the options from the best to the worst.

- 把偷东西的小动物赶出森林
  expel the animal from the forest whenever it steals other animals' property
- 教每个小动物保护自己的财物安全
  teach every animal to protect its property
- 不让偷东西的小动物参加集体活动
  do not allow a thief to join any forest activity
- 共同帮助生活里有困难的小动物
  help members of the community who are experiencing hardship

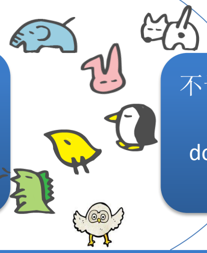

27

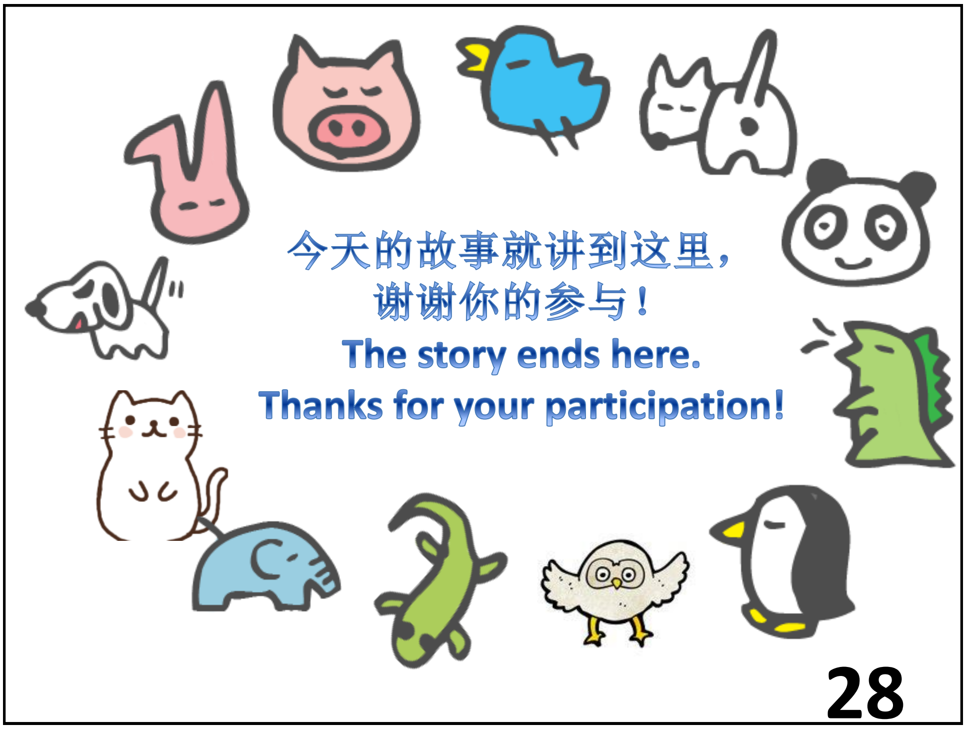

Supplement: Supplementary file 1 [file Image_1.pdf]
