# Supplementary material for: Young Children's Understanding of Restorative Justice
Source: Front Psychol. 2021 Sep 28;12:715279. doi: 10.3389/fpsyg.2021.715279 (PMC8506036; doi:10.3389/fpsyg.2021.715279)
Supplement: Supplementary file 2 [file Image_2.pdf]

小象和小猴子
的故事
A story of an elephant and a monkey

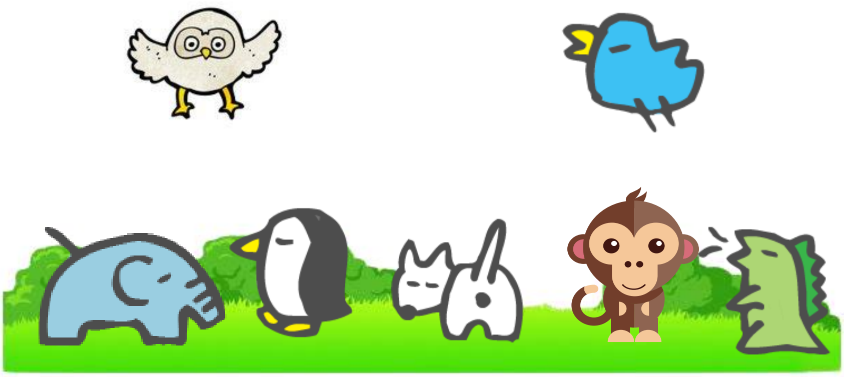

1

- 小象是农场里负责清洁的小动物，它可以用自己的鼻子吸走草地上的各种垃圾，把农场收拾得干干净净。
- A small elephant is a cleaner on a farm, and it can suck up rubbish with its nose and keep the farm clean and tidy.

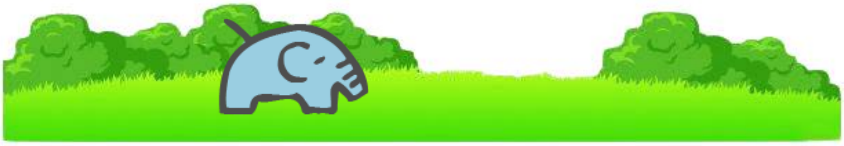

2

- 可是有一天，小象来到农场上，看到满地都是垃圾！它看了看周围，也不知道是谁做的。
- One day, when the elephant came to the farm, it found trash thrown everywhere. The elephant looked around, but it had no idea who had done this.

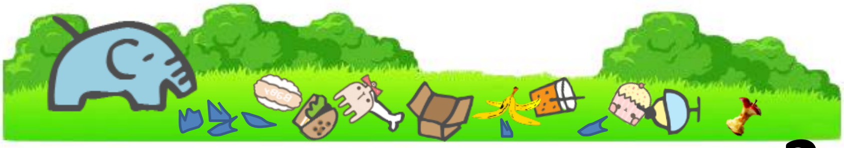

3

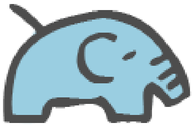

小象告诉了农场里的小动物，小动物们都不知道是谁扔的垃圾，大家都很惊慌，说："真气人，看来我们农场来了不爱干净的动物了！"

The elephant told this to the other animals on the farm, and they had no idea either. Everyone was anxious and said, "It is annoying; it seems that a messy animal has come to our farm!"

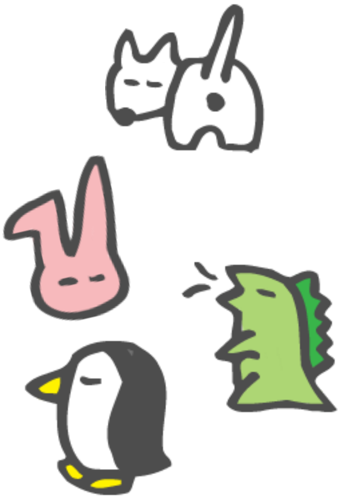

4

- 小象再次回到弄脏的农场，看著满地的垃圾，用自己的鼻子开始打扫起來。突然，地上的碎瓶子把小象的鼻子划了一個大口，小象的鼻子开始不停地流血了！

- The elephant went back to the farm and started to clean up the trash with its nose. All of a sudden, a piece of glass cut its nose, which started bleeding.

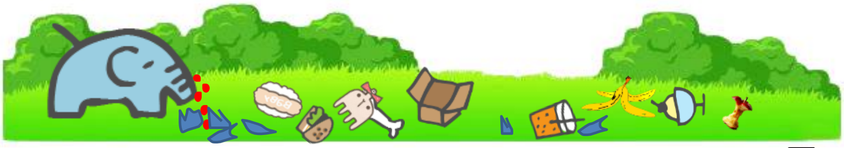

5

- 这个时候，小象看到小猴子草丛后面走了出来，一边吃着东西，一边扔着垃圾。小象生气地说道：“原来是这只小猴子，你扔的东西把我鼻子划伤了！”小猴子看到小象鼻子流血了，却说：“我不知道会划破你的鼻子呀！”

- At this moment, the elephant saw a monkey walking out from the bushes and throwing things around while eating its food. The elephant said angrily, “It’s you who threw the debris that cut my nose!” The monkey saw the elephant bleeding and said, “I didn’t know that it would cut your nose!”

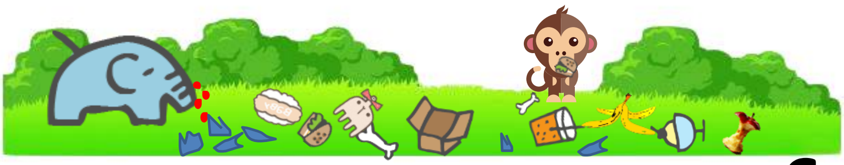

6

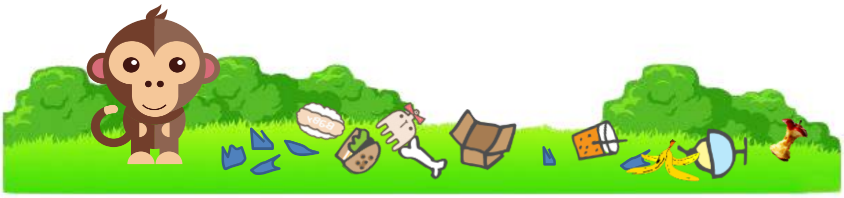

- 1. 小猴子扔东西对吗？
- Was it right or wrong for the monkey to throw the debris?
- Responses were scored on a 5-point scale ranging from 1 (very wrong) to 5 (very right).

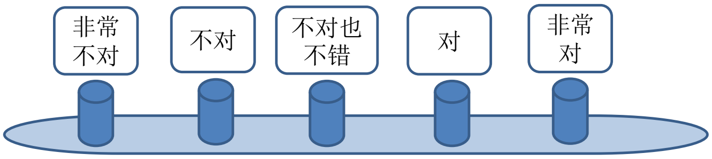

7

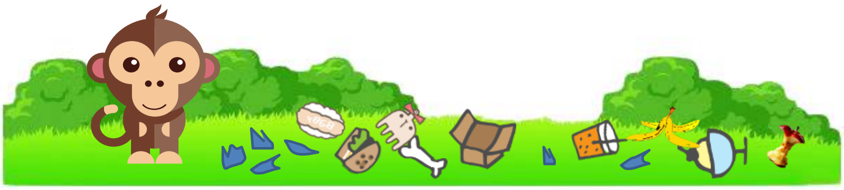

- 2. 小猴子是好还是坏呢？ Is the monkey good or bad? Responses were scored on a 5-point scale ranging from 1 (very bad) to 5 (very good).

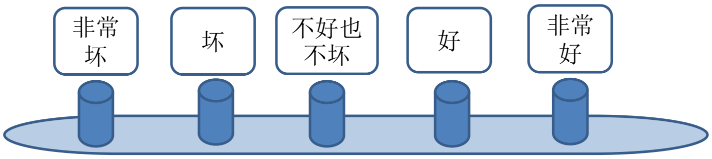

8

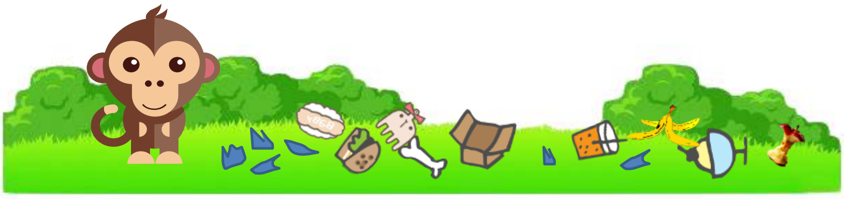

- 3. 那现在应该怎么办呢？
- What should be done now?

9

召开农场大会 Farm meeting

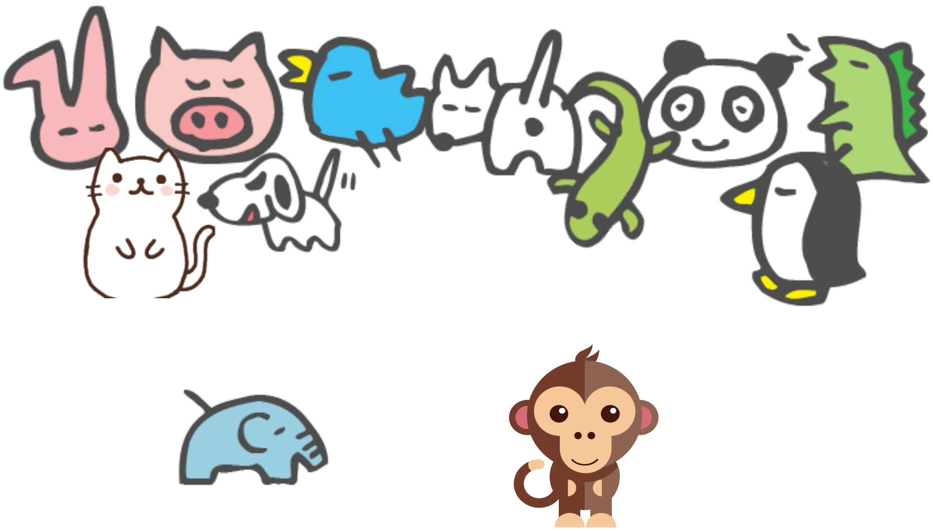

10

1. 小兔说：
A rabbit said,

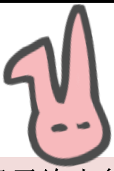

- “应该让小猴子给小象道歉。”
- “The monkey should apologize to the elephant.”

2. 小猫说：
A cat said,

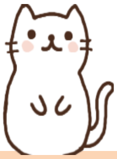

- “应该让小猴子帮小象把地清扫了。”
- “The monkey should clean the farm for the elephant.”

现在森林大会上四个小动物提出了这四个观点，我们要经过大家投票来决定哪一个最好。

Four animals proposed four views, we needed to vote to decide which one was the best.

3. 小狗说：
A dog said,

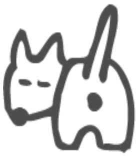

- “应该把小猴子关到笼子里。”
- “The monkey should be locked in a cage.”

4. 小鸟说：
A bird said,

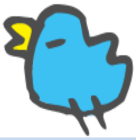

- “应该批评小猴子。”
- “The monkey should be criticized.”

11

投给小猫
Vote for the cat

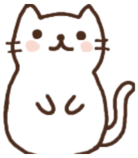

- “应该让小猴子帮小象把地清扫了。”
- “The monkey should clean the farm for the elephant.”

小猴子刚刚弄脏了5块地，那现在小猴子应该打扫多少块地呢？ The monkey polluted 5 pieces of lawn, so how many pieces of lawn should it clean?

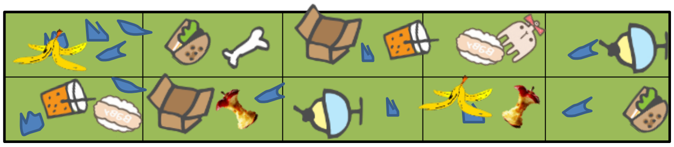

12

投给小狗
Vote for the dog

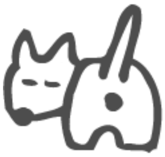

- “应该把小猴子关到笼子里。”
- “The monkey should be locked in a cage.”

应该把小猴子关多少天呢？How many days should the monkey get locked in the cage?

1天 10天 20天 30天 40天 50天 60天 70天 80天 90天 100天

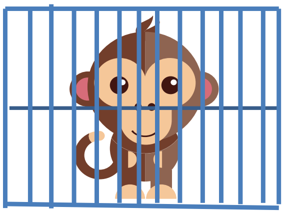

13

(Victim’s background)

- 农场大会上，大家发现，小象没有爸爸妈妈，它每天都在农场上认真工作，才能获得每天的食物。刚才，小猴子扔的垃圾把小象鼻子划伤了，小象现在不能工作了。

- In the farm meeting, we learned that the elephant is an orphan. It can only work for the farm to earn its living. The elephant has just been hurt by the debris and cannot work anymore.

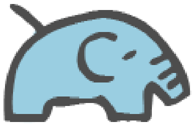

14

(Transgressor’s background)

- 农场大会上，大家发现，小猴子原来没有爸爸妈妈，没有人教过它吃完东西后要收拾垃圾。
- In the farm meeting, we learned that the monkey is an orphan. No one has ever taught it to clean up the debris left after eating a meal.

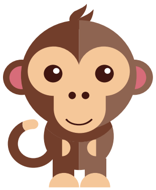

15

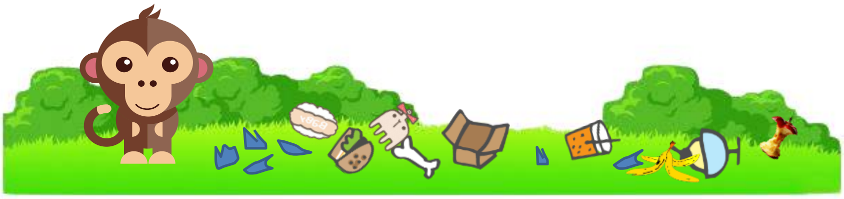

- 1. 现在你知道了小象（或小猴子）的故事，那么你觉得小猴子扔东西对吗？Now that you know the story of the elephant (or the monkey), was it right or wrong for the monkey to throw the debris?
- Responses were score on a 5-point scale ranging from 1 (very wrong) to 5 (very right).

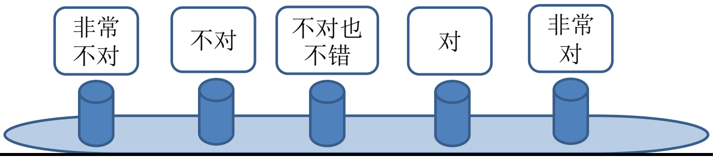

16

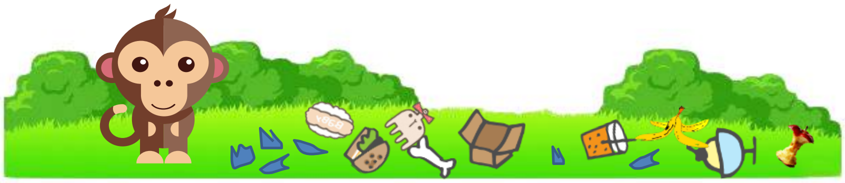

- 2. 小猴子是好还是坏呢？ Is the monkey good or bad? Responses were scored on a 5-point scale ranging from 1 (very bad) to 5 (very good).

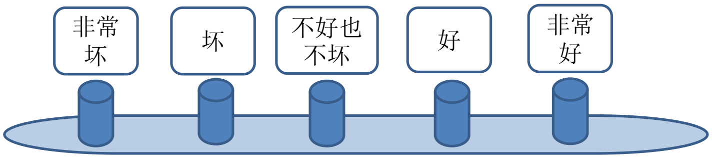

17

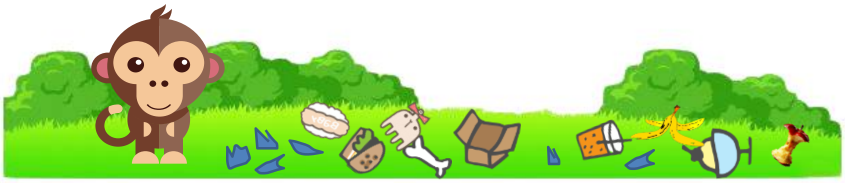

- 3. 那现在应该怎么办呢？
- What should be done now?

18

继续召开农场大会

Farm meeting continued…

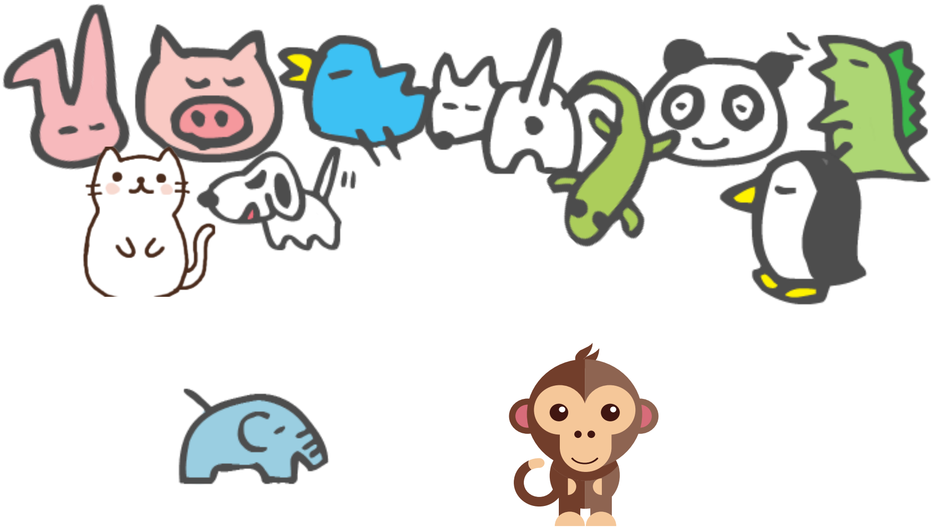

19

1. 小兔说：
A rabbit said,

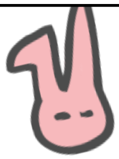

- “应该让小猴子给小象道歉。”
- “The monkey should apologize to the elephant.”

2. 小猫说：
A cat said,

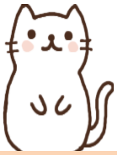

- “应该让小猴子帮小象把地清扫了。”
- “The monkey should clean the farm for the elephant.”

现在森林大会上四个小动物提出了这四个观点，我们要经过大家投票来决定哪一个最好。
Four animals proposed four views, we needed to vote to decide which one was the best.

3. 小狗说：
A dog said,

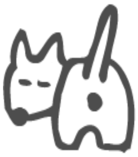

- “应该把小猴子关到笼子里。”
- “The monkey should be locked in a cage.”

4. 小鸟说：
A bird said,

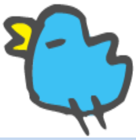

- “应该批评小猴子。”
- “The monkey should be criticized.”

20

投给小猫

Vote for the cat

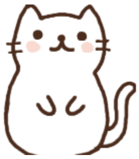

- “应该让小猴子帮小象把地清扫了。”
- “The monkey should clean the farm for the elephant.”

小猴子刚刚弄脏了5块地，那现在小猴子应该打扫多少块地呢？The monkey polluted 5 pieces of lawn, so how many pieces of lawn should it clean?

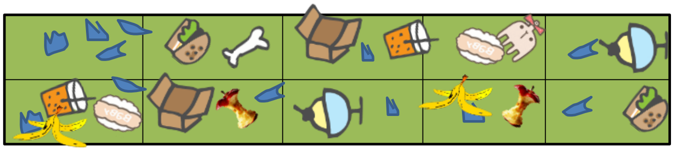

21

投给小狗

Vote for the dog

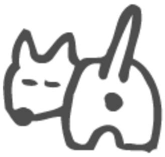

- “应该把小猴子关到笼子里。”
- “The monkey should be locked in a cage.”

应该把小猴子关多少天呢？How many days should the monkey get locked in the cage?

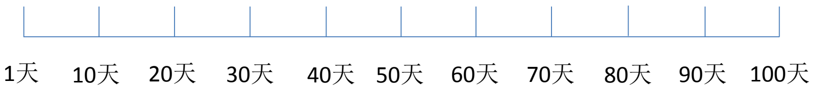

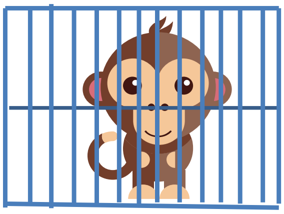

22

问题
Questions

- 小猴子的行为会影响到整个农场吗？
- Does the monkey's behavior impact the whole farm?

会 YES　　不会 NO

23

- 如果你选会的话，请问小猴子的行为对农场的影响有多大呢？How much does the monkey's behavior impact the farm？Responses were scored on a 4-point scale ranging from 1 (no impact) to 4 (very strong impact).

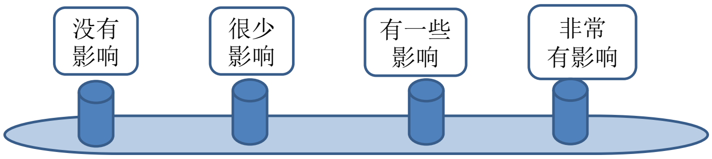

24

以前的农场是一个欢乐的大家庭，自从小猴子乱扔垃圾之后，大家也不愿意积极爱护农场卫生了，虽然小猴子最后打扫了5块地，也向小象道了歉，农场还是一天天脏了起来。

The farm used to be a happy family. Since the monkey polluted the lawn, the animals have become less willing to keep the farm clean. Although the monkey cleaned five pieces of lawn and apologized to the elephant, the farm is no longer a happy farm as before.

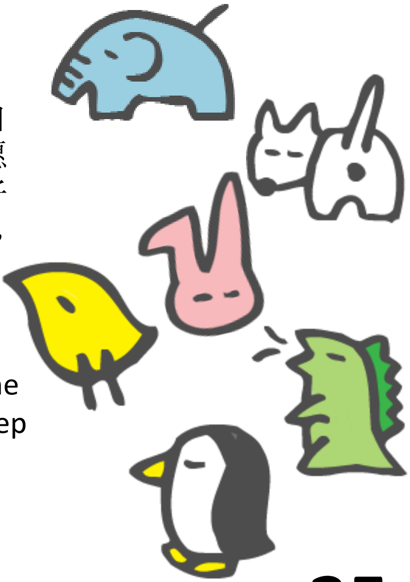

25

在这件事之后，作为农场的一员，我们应该怎么对待小象呢？
As members of the farm, how should we treat the elephant after this case? Please rank the options from the best to the worst.

帮小象找医生治疗鼻子 help the elephant find a doctor to heal its nose

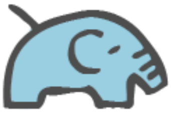

教它怎样在打扫卫生时保护自己 teach the elephant how to protect itself when doing cleaning work

和它做朋友，让它不要太难过 make friends with the elephant

26

在这件事之后，作为农场的一员，我们应该怎么对待小猴子呢？
As members of the farm, how should we treat the monkey after this case? Please rank the options from the best to the worst.

把它赶出农场
expel it from the community

教会它以后吃完东西要自己收拾垃圾
teach it how to clean up trash after a meal

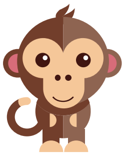

不让它和大家一起玩游戏
do not allow it to join any games with the other animals

让它作为农场监督员监督乱扔东西的行为
let it serve as the environment inspector for the farm

27

在这件事之后，作为农场的一员，我们社群应该做出什么改变呢？
As members of the farm, what changes should we make after this incident? Please rank the options from the best to the worst.

把破坏环境的小动物赶出农场
expel animals that throw trash around the farm

教育每个小动物不要丢垃圾，爱护环境 teach each animal to keep the environment clean

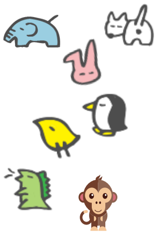

不让破坏环境的小动物参加集体活动 do not allow animals that pollute the environment to join any group activities

大家要共同承担起爱护环境的责任
ask every community member to shoulder the responsibility of environmental protection

28

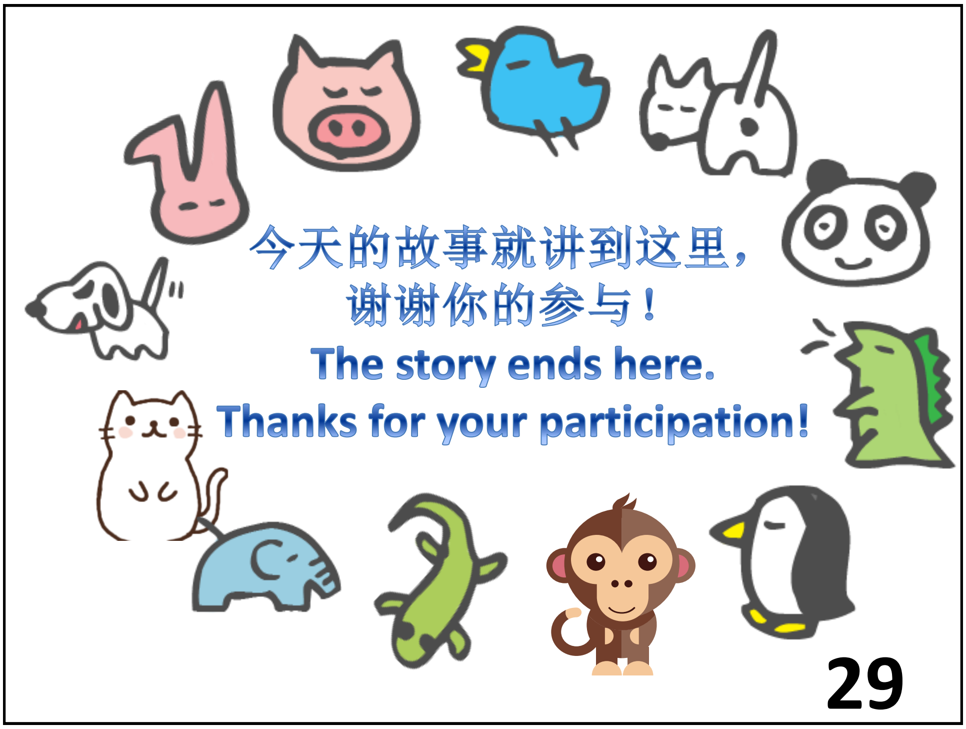
今天的故事就讲到这里，
谢谢你的参与！
The story ends here.
Thanks for your participation!
29
